# Cartilage thickness and bone shape variations as a function of sex, height, body mass, and age in young adult knees

**DOI:** 10.1038/s41598-022-15585-w

**Published:** 2022-07-09

**Authors:** Marco Tien-Yueh Schneider, Nynke Rooks, Thor Besier

**Affiliations:** 1 Auckland Bioengineering Institute, The University of Auckland, Level 6, 70 Symonds Street, Auckland, 1010 New Zealand; 2 Department of Engineering Science, The University of Auckland, Auckland, New Zealand

**Keywords:** Computational biology and bioinformatics, Anatomy, Biomarkers

## Abstract

The functional relationship between bone and cartilage is modulated by mechanical factors. Scarce data exist on the relationship between bone shape and the spatial distribution of cartilage thickness. The aim of the study was to characterise the coupled variation in knee bone morphology and cartilage thickness distributions in knees with healthy cartilage and investigate this relationship as a function of sex, height, body mass, and age. MR images of 51 knees from young adults (28.4 ± 4.1 years) were obtained from a previous study and used to train a statistical shape model of the femur, tibia, and patella and their cartilages. Five multiple linear regression models were fitted to characterise morphology as a function of sex, height, body mass, and age. A logistic regression classifier was fitted to characterise morphological differences between males and females, and tenfold cross-validation was performed to evaluate the models’ performance. Our results showed that cartilage thickness and its distribution were coupled to bone morphology. The first five shape modes captured over 90% of the variance and described coupled changes to the bone and spatial distribution of cartilage thickness. Mode 1 (size) was correlated to sex (p < 0.001) and height (p < 0.0001). Mode 2 (aspect ratio) was also correlated to sex (p = 0.006) and height (p = 0.017). Mode 4 (condylar depth) was correlated to sex only (p = 0.024). A logistic regression model trained on modes 1, 2, and 4 could classify sex with an accuracy of 92.2% (95% CI [81.1%, 97.8%]). No other modes were influenced by sex, height, body mass, or age. This study demonstrated the coupled relationship between bone and cartilage, showing that cartilage is thicker with increased bone size, diaphysis size, and decreased femoral skew. Our results show that sex and height influence bone shape and the spatial distribution of cartilage thickness in a healthy young adult population, but body mass and age do not.

## Introduction

The functional relationship between bone and cartilage is complex and is modulated by mechanical factors introduced by loading and motion^1^. Mature cartilage has a location-dependent histomorphology that is developed in response to its specific loading history^1^. The mechanical stresses and strains experienced by the cartilage over time influence the morphology and material properties of the tissue. Conversely, the morphology of bone and cartilage influences the instantaneous stresses and strains experienced in the joint^2^. Bone size and shape can affect the contact area and the lines of action of the muscles and supporting ligaments crossing the joint^3^, while cartilage thickness influences the mechanical properties of cartilage^4^ and the stresses and strains experienced in the tissue^2,5,6^. Characterising the relationship between bone and cartilage morphology in vivo is important to understand the knee joint’s functional anatomy and pathology.

Knee bone morphology has been characterised as a function of sex^7,8^, osteoarthritis (OA)^9–12^, and knee ligament injury^13^. Studies have also quantified cartilage thickness across individuals^5,14–16^ and found that cartilage is generally thicker where cartilage stresses are high^6,17^. Males appear to have greater volume of knee cartilage compared to females^18,19^, and cartilage morphology is in part mediated by size and body mass of the individual^15,17,18^. However, these studies have analysed cartilage and bone morphology in isolation or have simplified cartilage morphology to a singular value (such as cartilage volume or mean thickness), and thus, do not capture the complexity and nuance of the coupled variation in cartilage thickness distribution and bone morphology in three dimensions. This information may have important implications for understanding the onset of pathology such as OA, the development of surrogate and predictive models, and the design of implants.

Statistical shape modelling (SSM) characterises complex 3D morphologies by decomposing shape features into a set of statistically significant principal components that describe the main variations in the population^20,21^. The shape features can be extended to include scalar field values (such as cartilage thickness, and bone mineral density) to explore shape-field relationships. The principal components, or modes, can then be used to visualize variations and correlate to parameters such as patient demographics to quantitatively examine differences between cohorts^10,22–24^. To the best of our knowledge, statistical shape models have not been used to characterise the coupled morphological variations of adult knee bones and their cartilage thickness distribution in relation to subject demographics.

The purpose of this study was to develop a statistical shape and cartilage thickness field model of the adult knee, consisting of the femur, tibia, patella and their cartilages, to characterise the coupled variation in bone morphology and cartilage thickness as a function of sex, height, body mass, and age, in a cohort of 51 knees with healthy cartilage.

## Methods

**Figure 1:**
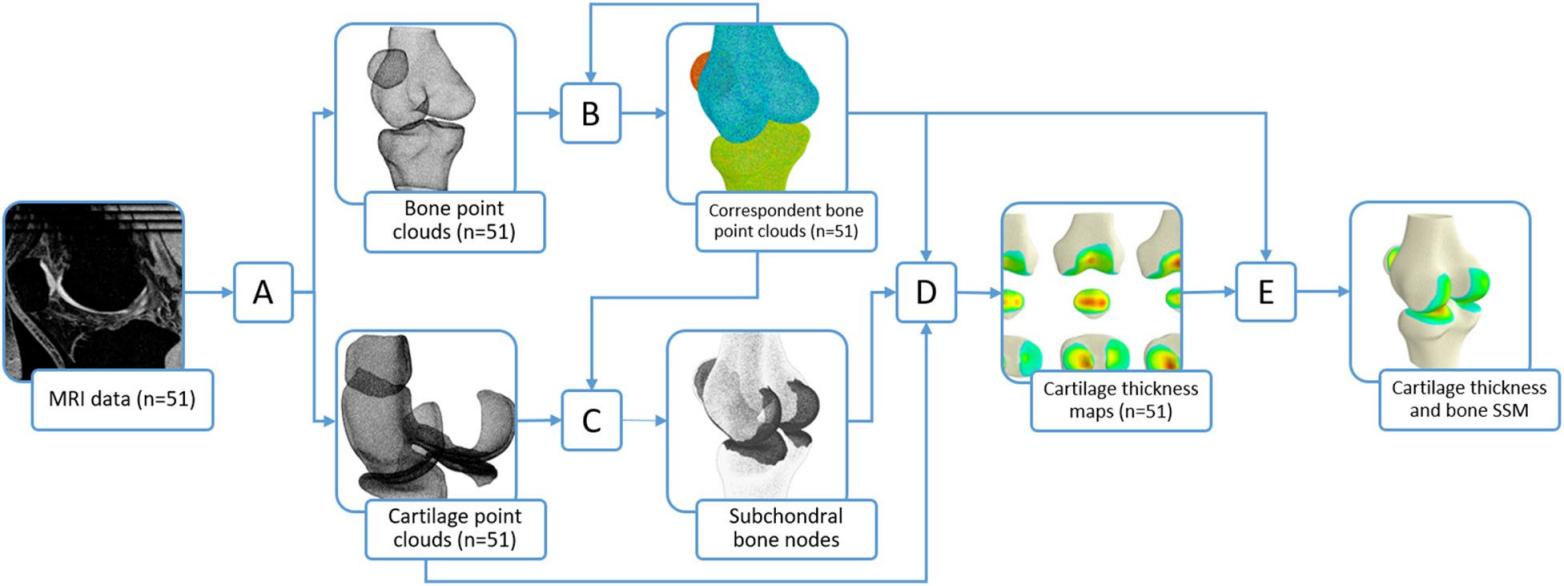
Schematic of workflow to generate cartilage thickness field and bone shape model. MR images (n = 51) of the knee were segmented, processed in MAPClient, and resampled (**A**) to produce bone and cartilage point clouds. Parametric or correspondent bone point clouds were obtained via an iterative fitting process (**B**). Subchondral bone node numbers on these correspondent point clouds were found by combining node numbers obtained from all subjects using a closest-point algorithm (**C**). Cartilage thickness maps were calculated (**D**) by computing the magnitude of the projection of the closest articular cartilage point to the normal vector of each subchondral bone node. Principal component analysis (**E**) was performed on features consisting of the corresponding nodal coordinates of the bone and the cartilage thickness per subchondral node to produce a statistical model of the cartilage thickness and bone shape.

MR images (n = 51) of adult knees (28.4 ± 4.1 years, Table 1) were obtained from an ongoing study on patellofemoral pain^16,25,26^, where all participants were screened by a radiologist to ensure they had no cartilage damage or degenerative changes. All participants were advised on all aspects of the study and analysis of data before informed consent was obtained. Permission was obtained to analyse the data in this study. Ethics approval for this study (Reference #3346) was granted by the Stanford University Institutional Review Board, and the procedures followed were in accordance with the ethical standards of the responsible committee on human experimentation (institutional and national) and with the Helsinki Declaration of 1975, as revised in 2000. All methods were carried out in accordance with relevant guidelines and regulations. The bones, including the femur, patella, tibia, and their corresponding cartilages, including both the subchondral and articular surfaces, were manually segmented in Stradwin^27^ to produce triangulated surface meshes and point clouds (Fig. 1A). To correct for the varying field of view (FOV) in the imaging data, MAPClient^28^ was used to fit an existing statistical shape model (SSM)^8^, trained on a CT dataset (n = 204) from the Victoria Institute of Forensic Medicine, to the femurs and tibias (Fig. 1A). This fit consisted of an SSM fit followed with a local nodal fit to a root-mean-squared (RMS) error < 1 mm. The femurs were then cropped at a height equal to the epicondylar width and the tibias were cropped at a height equal to the width of the tibial plateau.

**Table 1:** Subject demographics.

	Number	Age (years)	Height (cm)	Body mass (kg)
Total	51	28.4 ± 4.1	172.0 ± 8.5	65.6 ± 10.2
Females	30	28.2 ± 4.6	167.5 ± 5.9	60.6 ± 7.7
Males	21	29.3 ± 3.7	178.4 ± 7.4	72.7 ± 9.0

A published method^10,22,24^ was adapted to maximise nodal correspondence in the bone point clouds and create a training set of parametric point clouds (Fig. 1B). One set of bone point clouds were selected and resampled in Meshlab^29^ to generate template point clouds with mean nearest neighbour distance of 0.5 mm and mesh density of ~ 2.6 points per mm^2^ for the femur, tibia, and patella (Fig. 1A). The template point clouds were iteratively fitted to the training set data via a series of coarse to fine fits using adaptive radial basis function fitting to generate a training set of maximally correspondent points^30^. Rigid alignment of the training set data to the templates was achieved by minimising the least squared distances of corresponding nodes. Principal component analysis (PCA) was performed on the correspondent nodes to generate an intermediate statistical shape model of the bones^31^. This process was repeated with the shape model as the template until the correspondent RMS error between the point cloud in the current and previous iteration was less than 0.01 mm.

The cartilage point clouds were resampled (Fig. 1A) to a density of ~ 16 points per mm^2^, resulting in ~ 200,000 points for the femoral cartilage, ~ 50,000 points for the patellar cartilage, and ~ 50,000 points each for the lateral and medial tibial cartilages. The cartilage point clouds for each subject in the training set were overlaid with their corresponding parametric bone point clouds. A closest point algorithm was used to identify bone nodes that were closest to the subchondral cartilage layer (Fig. 1C). These bone nodes, of all subjects, were combined to produce a list of subchondral bone nodes shared by the training set.

The cartilage thickness was then calculated for each subchondral bone node (Fig. 1D)^32^. The closest articular cartilage point to the normal of each subchondral bone node was projected onto the normal vector. The magnitude from the subchondral bone node to this projection was calculated to obtain the thickness at that subchondral bone node. This calculation was performed on the femur, tibia, and patella for all subjects in the training set (n = 51) to obtain correspondent maps of cartilage thickness.

Correspondent nodes and cartilage thickness at each node were then analysed using PCA. A 2D matrix was constructed with n-rows (n = 51 independent observations) and m-columns of features, consisting of mean centred nodal coordinates and thickness values. The matrix was normalised by dividing all columns by its standard deviation. PCA was performed on the resulting matrix to produce a statistical shape and field model (SSFM) of bone shape and cartilage thickness (Fig. 1E).

The SSFM scores of the first *n* modes, such that the cumulative variation captured was over 90%, were used for statistical analysis. Multiple linear regression was used to investigate the influence of sex, height, body mass, and age on the SSFM scores.

A multivariable logistic regression analysis^33^ was performed on the scores of the SSFM to characterise the coupled morphological differences in bone shape and cartilage thickness distribution between males and females. Principal components were added in a stepwise fashion until no more statistically significant improvement of the fit of the model was observed. Tenfold stratified cross-validation was performed to evaluate the models performance.

## Results

**Figure 2:**
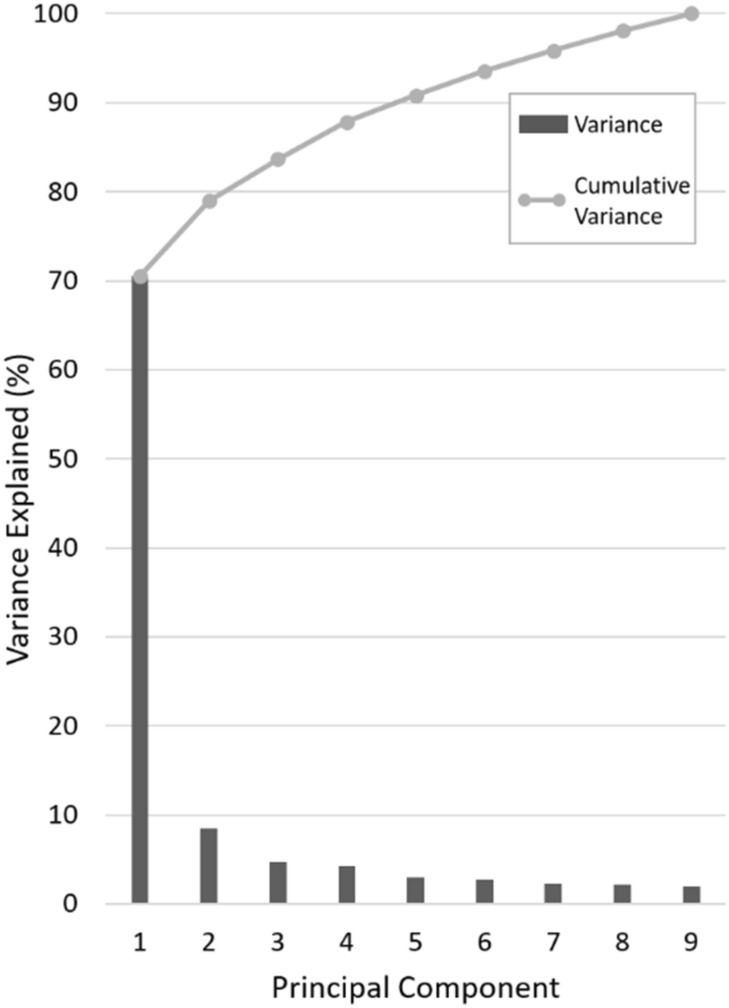
Variation and cumulative variation represented by principal component number for the statistical bone shape and cartilage thickness model.

The first five principal components (or modes) of the SSFM of bone shape and cartilage thickness captured over 90%, of the variation in the morphology in the training set (Fig. 2).

**Figure 3:**
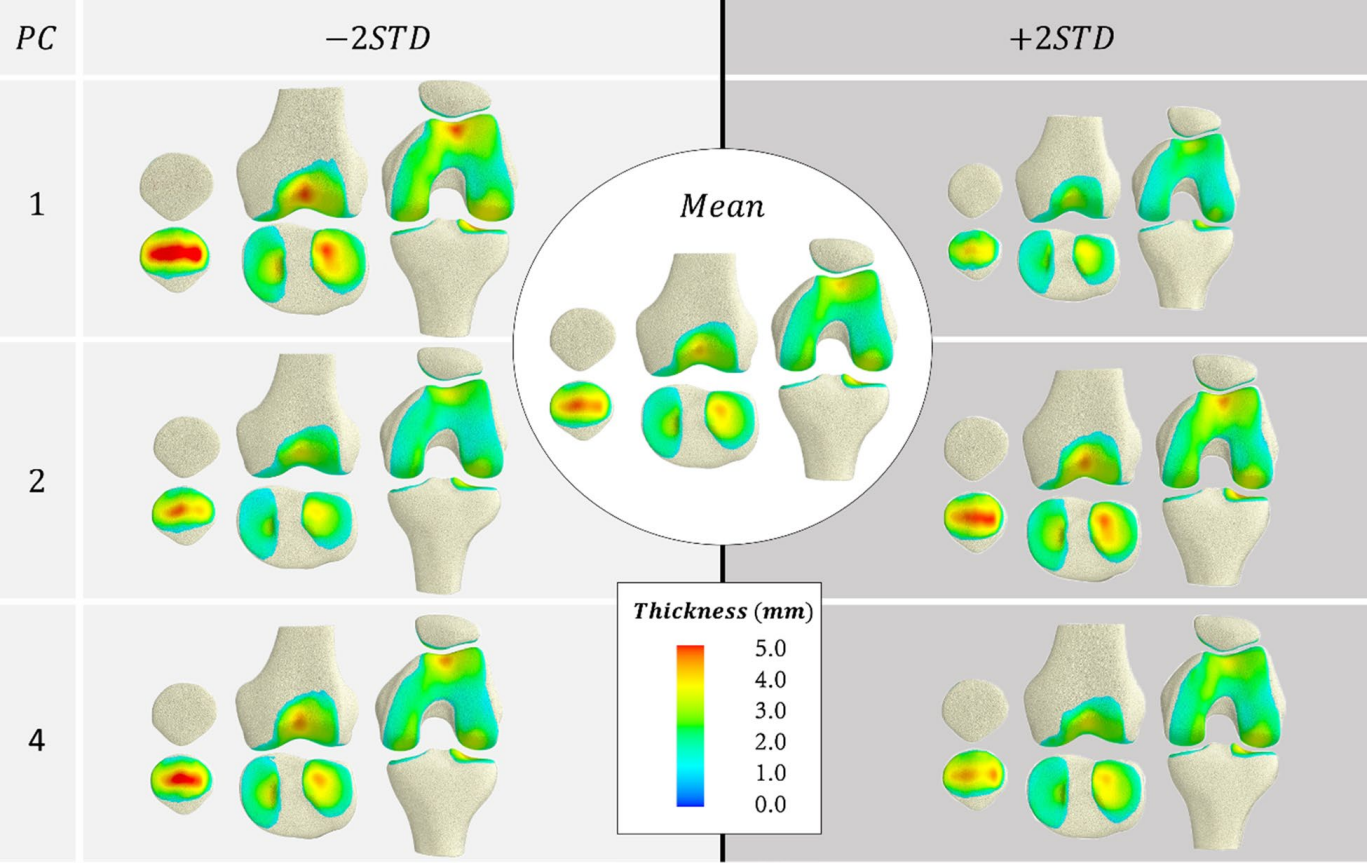
Coupled variations in bone shape and cartilage thickness of the SSFM showing principal components that were influenced by sex and height (PC1 and PC2), or sex (PC4).

**Figure 4:**
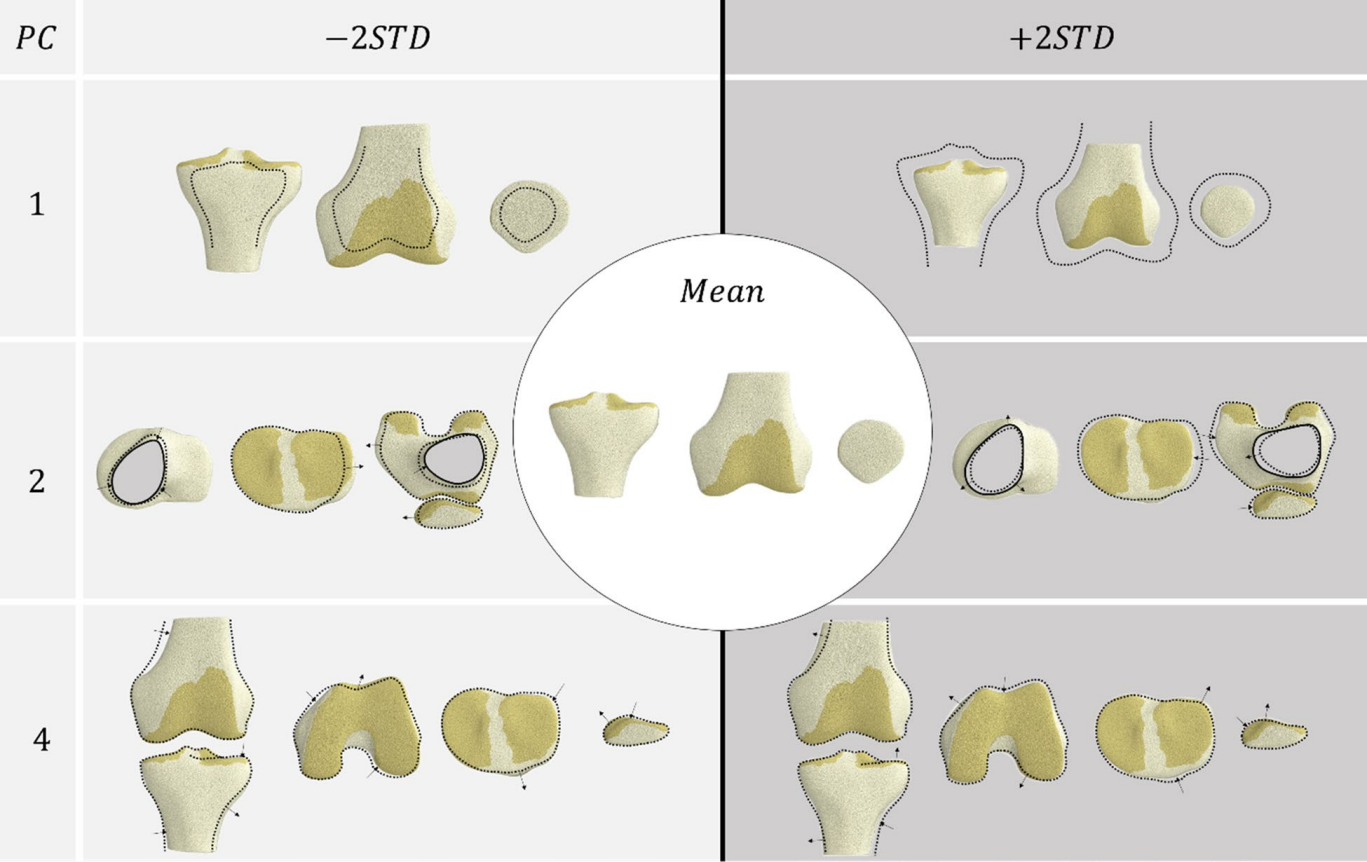
Coupled variations in bone shape of the SSFM showing principal components that were influenced by sex and height (PC1 and PC2) or sex (PC4).

Multiple linear regression models were fitted for the first five modes of the SSFM (Table 2). The first mode(size) was strongly influenced by sex (p < 0.001) and height (p < 0.0001). The second mode was also influenced by sex (p = 0.006) and height (p = 0.017). The fourth mode was influenced by sex (p = 0.024). These modes are visualised in Figs. 3 and 4. No other modes were influenced by sex, height, body mass, or age.

**Table 2:** Standardised coefficients (beta) of multiple linear regression for modes in the SSFM.

Mode	**1**	**2**	**3**	4	5
Sex	− 0.323**	− 0.500**	0.0911	− 0.415*	0.296
Height	− 0.508**	0.548*	− 0.0925	− 0.0619	0.0131
Mass	− 0.162	− 0.226	− 0.2206	0.320	-0.322
Age	− 0.0322	0.121	0.114	0.162	0.196
R^2^	0.795	0.200	0.0626	0.159	0.0997

*^,^**Significance at the 95% and 99% level, respectively.

The regions with the thickest cartilage in the femur were located in the trochlear groove, the femoral condyles, and along the medial condyle (Fig. 3). In the tibia, the thickest cartilage was found in the lateral tibial plateau. In the patella, the thickest cartilage was generally located centrally with some variations where the distribution bifurcated across the patella’s vertical ridge.

The first mode qualitatively explained differences in the overall size of the knee bones as well as the overall thickness of the cartilage (Fig. 3). From this model, we observed that bone size was coupled to cartilage thickness, with larger bones having thicker cartilage. Male sex and tall height were correlated with larger bones and thicker cartilage (more negative PC1) (Table 2).

The second mode described coupled changes associated with aspect ratio or width along the mediolateral (ML) direction of the bones and cartilage. In the bones (Fig. 4), we observed coupled changes in the size of the diaphysis relative to the ML width of the epiphyses in both the femur and tibia. In the tibia, we also saw a widening of the tibial plateau with decreased diaphysis size. This widening was accompanied with a wider distal femur and patella. Additionally, this mode captured changes in the anterior–posterior thickness of the patella, which was lower with decreased diaphysis size. In the cartilage, we observed a number of coupled changes across the bones (Fig. 3), including thinning of cartilage in both the femur and tibia with decreased diaphysis size, and a proximal–distal shift in the location of the thickest cartilage in both the patella and trochlea cartilage. Furthermore, thinning of the trochlear cartilage on the femur was coupled with thicker patellar cartilage on the lateral side of the vertical ridge and thinner cartilage on the medial side. Male sex and shorter height were correlated with high aspect ratio (more negative PC2) (Table 2).

In the bone, the fourth mode describes the depth of the femoral condyle in the anteroposterior (AP) direction. It also describes changes to the angle of the femoral shaft axis to the epicondylar axis (femoral shaft angle) and the mediolateral tilt of the tibial plateau. A deeper distal femur was accompanied with a larger femoral shaft angle and a flatter tibial plateau (Fig. 4). In addition, a deeper distal femur was accompanied with a less pronounced ridge located on the medial side of the patella. A more pronounced ridge was coupled with a bifurcation in cartilage thickness across the patellar ridge. This increased condylar depth was accompanied with thicker cartilage throughout and a more superiorly located cartilage boundary in the trochlea (Fig. 3). Male sex was correlated with greater condylar depth (more negative PC4) (Table 2).

**Figure 5:**
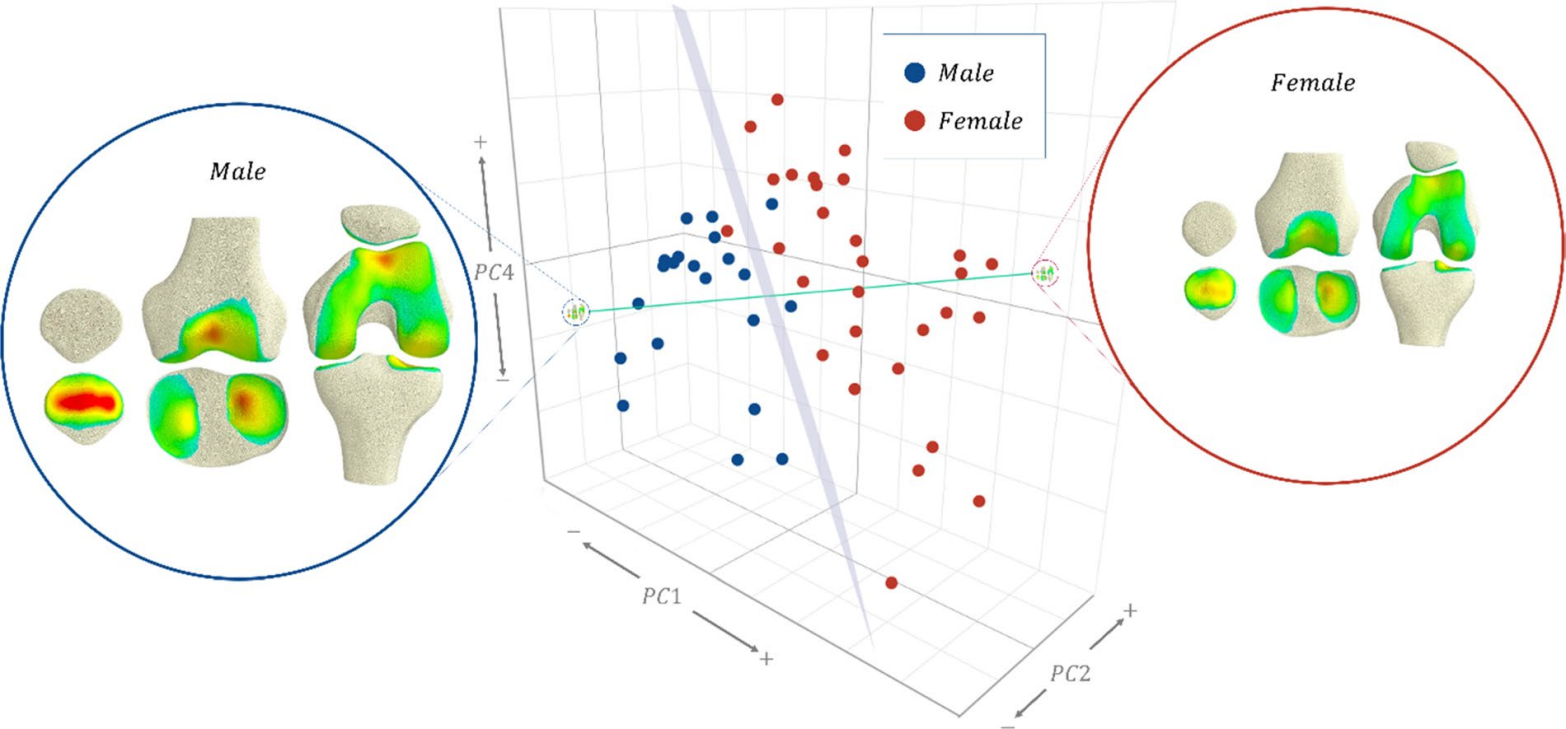
3D scatter of principal components 1, 2, and 4, of the training set by sex, showing the decision boundary plane (grey) of the logistic regression model, and, a vector (green) that passes through the average male and female knee.

The logistic regression model trained on principal components 1, 2 and 4 (Fig. 5) of the SSFM scores characterised the morphology of the bone and cartilage between males and females to an accuracy of 94.1% within the training set. Cross-validation of this model yielded a classification accuracy of 92.2% (95% CI [81.1%, 97.8%]) and an area under the curve (AUC) of 94.1%. The decision boundary (Fig. 5), shows that females had generally smaller bones (positive PC1) accompanied with lower aspect ratios (positive PC2), steeper mediolateral tibial tilt, more pronounced patellar ridge, and thinner distal femur in the AP direction (positive PC4).

## Discussion

The purpose of this study was to develop a statistical shape and field model of the femur, tibia, patella, and their cartilages to characterise the coupled variation in bone morphology and cartilage thickness in a cohort of healthy knees. The bone shape and cartilage field model captured over 90% of the variance with the first five modes. This model was interrogated to explore the effects of sex, height, body mass, and age, on the morphology of the bones and cartilage thickness distribution, and finally used to characterise the morphological differences between males and females. We found that sex and height strongly influenced knee joint morphology, while body mass and age did not influence morphology in this young adult cohort.

Our results showed that cartilage thickness and its distribution was coupled to bone morphology, including both size (mode 1) and shape variations (mode 2 onwards).

The greatest influence on the overall cartilage thickness was bone size, where larger bones displayed thicker cartilage (Fig. 3, mode 1). Although this seems intuitive, studies across species have shown that cartilage thickness does not necessarily scale with size. For example, the equine cartilage is thinner than human cartilage in smaller equivalent joints^34,35^, despite equine cartilage experiencing much higher stresses than humans^36–39^. Within the lower limb joints of humans, Shepherd and Seedhom^40^ hypothesised that cartilage thickness is a function of joint congruency, due to the hip and ankle having thinner cartilage than the knee in their sample of 11 cadavers. This is believed to be related to how much stress the cartilage needs to bear^6,17^. We expect an increase in body mass with bone size to result in increased stress that requires thicker cartilage to bear. Studies have also reported a correlation between anthropomorphic measurements such as height and mass to mean cartilage thickness^15,17,40^, implying a relationship between bone size, stress, and cartilage thickness. Our results confirm a part of this finding in a larger sample (n = 51), where height correlated to increased overall cartilage thickness (mode 1). Previous studies indicated that mass only described some of the variability in cartilage thickness^15,17,40^. However, our results show that mass was not correlated with any of the modes of variation. This is unexpected, since mass is typically a predictor of muscle volume and thus, muscle forces, which dominate joint contact forces and are related to the load history during growth and adolescence when the bone and cartilage is undergoing a modelling process^41^. The literature has shown that incorporating information beyond mass, such as height, can enable better predictions of joint moments during locomotion and muscle volumes in the lower limb^42^. However, we found that none of the modes investigated were correlated with mass, mass × height, or BMI when height was included in the model.

Our model showed that cartilage thickness was coupled to bone shape variations. Knees with higher aspect ratio and smaller diaphyses (mode 2) possessed thinner cartilage overall and the location of the thickest cartilage in the patella and trochlea shifted proximally, whereas knees with lower aspect ratio and larger diaphyses possessed thicker cartilage. Increased diaphysis size increases the bone section modulus which typically results from increased load experienced during growth and development^43^. This increased load bearing may explain why this feature is accompanied with thicker cartilage.

The mediolateral slope of the tibial plateau and the angle of the femoral shaft axis were also coupled (mode 4) where a larger angle was accompanied with thicker distal femur in the AP direction. Recent studies have reported that a high Q-angle (similar to the femoral shaft axis angle) is negatively correlated with cartilage thickness^44^, especially in the lateral compartment^45^. Kusiak and Kawczyński^45^ hypothesised that this is due to increased compression imposed by the patellar cartilage on this condyle. Our results do not appear to support this hypothesis, but instead suggest that the patellar and trochlear cartilages get thicker with increased femoral shaft axis angle. Without knowledge of the muscle force distribution on the patella and the contact mechanics of the patellofemoral articulation, it is difficult to draw any conclusions regarding patellofemoral joint mechanobiology. Further modelling work might uncover these form-function relationships and sex differences.

Sexual dimorphism was present in three modes of the model (Fig. 5). Size (mode 1), aspect ratio (mode 2) and condylar depth (mode 4) of both the bone and cartilage were important for classifying sex. On average, this meant that females presented a smaller joint with thinner cartilage, with a lower aspect ratio and shallower distal femur compared to males. This is somewhat expected and conforms with previous studies that have demonstrated sexual dimorphism in bone morphology^10,46^. Kim et al.^46^ showed that four key morphometric measures including condylar width, width of the medial condyle, and the depths of the lateral condyle and intercondylar notch were important for sex classification. These measures were captured in the modes of our model, for example, size and aspect ratio capture all four key measures identified, while condylar depth captures the depth of the lateral condyle. Prior studies have also reported differences in cartilage volume between males and females, where males had larger cartilage volumes than females^16–19,47^. Our results add to these previous findings by giving further clarity as to how the spatial distribution of the cartilage thickness varies in the population along with variations to bone morphology. We expect further modelling work to reveal how cartilage stress is maintained across different bone and cartilage morphologies.

There are many implications of this work. It is important to consider both the cartilage morphology as well as the bone morphology as they both influence the mechanics. This is particularly important when considering joint contact models as the cartilage thickness influences the mechanical response of the cartilage and the internal stresses experienced by the tissue. In implant design, the articulating geometry needs to be considered, not just the subchondral bone. This method can be extended to include an OA cohort to compare the coupled changes in bone and cartilage morphology as a function of OA progression.

There are several limitations in this work that should be acknowledged. Firstly, the conclusions drawn in this paper are limited to knees with healthy cartilage, as determined by a musculoskeletal radiologist as part of the inclusion criteria from the original experimental study^16^. Some of these participants had patellofemoral pain, which may influence the results. Across the age-range of our study cohort we did not observe any morphological differences due to age. It is possible that widening the age-range of the study cohort would lead to detection of age-related differences. We expect to observe age-related morphological differences in the bone and cartilage in an adolescent population where growth and development occurs, and in an older population where OA degeneration may be occurring. Our methodology normalised for bone orientation, a factor that may influence the interaction between bones and hence the cartilage thickness. As the scope of this study was to characterise the coupled variation in bone morphology and cartilage thickness, we did not investigate how these relationships are affected when normalised for bone size or maximum cartilage thickness. Lastly, as with any investigation of bone and cartilage morphology using medical imaging data, our results are subject to manual segmentation errors which are typically < 0.5 mm for knee joint structures^32^.

In conclusion, we have characterised and described the coupled variation in bone morphology and cartilage thickness in a cohort of 51 young adult knees. We observed that cartilage is thicker with increased bone size, diaphysis size, and condylar depth. Sexual dimorphism was present in these three modes which may be used to understand the dimorphism that exists in OA, knee ligament injuries, and patellofemoral pain. Lastly, we found that bone morphology and cartilage thickness were strongly correlated with height but not body mass or age.

## Data Availability

The data that support the findings of this study are openly available in SimTK.org Anatomical Knee at https://simtk.org/projects/anatomicalknee^48^.
